# Lumbopelvic Fixation for Sacral Insufficiency Fracture Presenting with Sphincter Dysfunction

**DOI:** 10.1155/2019/9097876

**Published:** 2019-04-07

**Authors:** Satoshi Maki, Kaito Nakamura, Tomonori Yamauchi, Takeshi Suzuki, Manato Horii, Koui Kawamura, Masaaki Aramomi, Hiroshi Sugiyama, Seiji Ohtori

**Affiliations:** ^1^Department of Orthopaedic Surgery, Asahi General Hospital, i1326 Asahi City, Chiba 289-2511, Japan; ^2^Department of Orthopaedic Surgery, Chiba University, Graduate School of Medicine, 1-8-1 Inohana Chuou-ku, Chiba City, Chiba 260-8670, Japan; ^3^Research Center for Allergy and Clinical Immunology, Asahi General Hospital, i1326 Asahi City, Chiba 289-2511, Japan

## Abstract

Sacral insufficiency fractures (SIFs) are common in the elderly. In patients with SIF, objective neurological abnormalities such as sphincter dysfunction or leg paresthesia are uncommon. We present a case of SIF accompanied by spinopelvic dissociation with late neurological compromise treated by spinopelvic fixation. A 61-year-old woman presented to our hospital with low back pain without obvious trauma history. She had a past history of eosinophilic granulomatosis with polyangiitis and treatment with steroids. Her low back pain became worse, and she started to have radiating left posterior thigh pain and motor weakness in the left ankle and both great toes with symptoms of stress urinary incontinence, constipation, and loss of anal sensation. Magnetic resonance imaging revealed an H-shaped sacrum fracture. We attributed the neurological symptoms to unstable SIF and performed lumbopelvic fixation. After the surgery, her leg pain and symptoms of stress urinary incontinence improved markedly, as did anal sensation. At a 6-month follow-up, the patient reported no low back pain and she was walking independently without pelvic complaints. CT showed bone union was achieved. Even minimally displaced SIF in patients with osteoporosis can be a cause of bowel and bladder disturbance. Lumbopelvic fixation is a treatment option for SIF with spinopelvic dissociation presenting neurological deficit.

## 1. Introduction

Increasing numbers of fragility fractures of the pelvis have been reported with increasingly aging populations [1]. Sacral insufficiency fracture (SIF) accounts for more than half of all fragility fractures of the pelvic ring [2]. The risk factors for SIF are reported as osteoporosis, pelvic radiation therapy, rheumatoid arthritis, long-term corticosteroid therapy, and being postmenopausal [3]. Although neurological symptoms of radiculopathy and myelopathy are present in up to 70% of patients with SIF, objective neurological abnormalities such as sphincter dysfunction or leg paresthesia are uncommon (2%-14%) [4]. Here, we present, to our knowledge, the first report of a case of SIF in a patient presenting late neurological compromise that was treated with spinopelvic fixation.

## 2. Case Presentation

A 61-year-old woman presented to our hospital with low back pain. Trauma history was vague except for falling 40 cm from a bed a month earlier. She had a past history of eosinophilic granulomatosis with polyangiitis and was treated with prednisolone (40 mg/day) 3 months before presenting at our hospital. Her bone mineral density in the hip had a T-score of −4.7, and bisphosphonate and vitamin D were initiated to treat osteoporosis. She underwent computed tomography (CT) and was diagnosed as having a left vertical sacral fracture and treated conservatively. Three weeks after her initial diagnosis of sacral fracture, her low back pain worsened and she started to have radiating left posterior thigh pain with symptoms of stress urinary incontinence, constipation, and loss of anal sensation.

**Figure 1:**
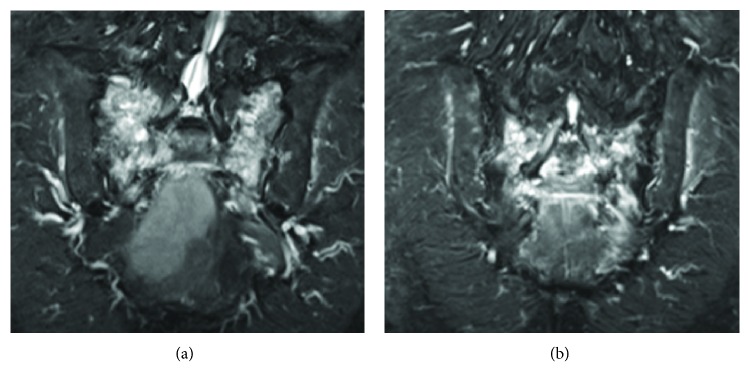
Lumbosacral magnetic resonance imaging with short inversion time inversion recovery (STIR) sequence shows an H-shaped sacrum fracture. Bilateral vertical fractures of the lateral mass of the sacrum and horizontal fracture at the level of the S2 neural foramina are visible.

**Figure 2:**
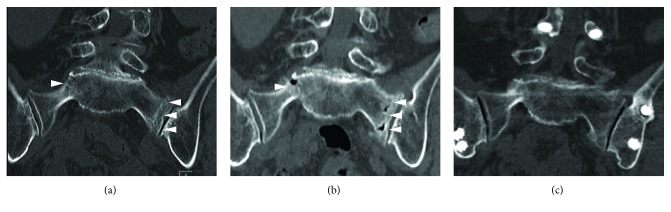
Coronal sacral CT images obtained at the first visit (a), admission (b), and final follow-up (c). The initial CT revealed nondisplaced bilateral fracture of the lateral mass of the sacrum (white arrow head). At the time of admission, CT showed slightly aggravated displacement of the fracture (white arrow head). CT at the final follow-up demonstrated bony union of the sacral lateral mass fracture. CT: computed tomography.

On examination, she had weakness of dorsiflexion of her left ankle and flexion of both great toes, in which the muscular power was grade 3/5 and 4/5, respectively, as measured with manual muscle testing (MMT). Sensation to pinprick on her S2 receptive field was impaired. Lumbosacral magnetic resonance imaging using a short inversion time inversion recovery (STIR) sequence revealed an H-shaped fracture in the sacrum, but there were no abnormal findings such as spinal stenosis in her lumbar spine potentially explaining for her neurological symptoms (Figure 1). Repeated CT demonstrated slightly aggravated displacement of the sacral fracture compared with previous CT (Figure 2).

**Figure 3:**
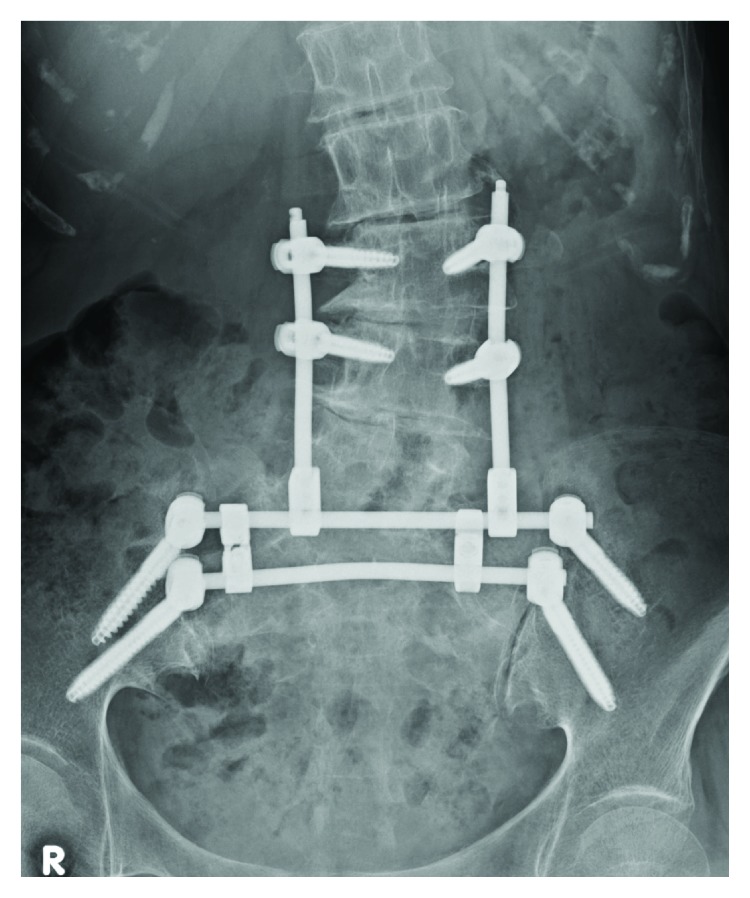
Postoperative anteroposterior radiograph. Percutaneous pedicle screws were inserted in L3 and L4, and 2 iliac screws were inserted on each side. Bilateral iliac screws were connected by 2 transverse rods and a crosslink. The crossing points of rods for lumbar pedicle screws and the cranial transverse rod for iliac screws were connected by rod connectors, completing lumbopelvic fixation.

We consulted a urologist, and the patient was diagnosed as having stress incontinence because of poor function of pelvic floor muscles or the sphincter. However, we speculate that both the weakness of dorsiflexion of the ankle and great toes and loss of anal sensation could be attributed to SIF and recommended that she undergo surgery. She underwent lumbopelvic fixation from L3 to the ilium using a minimally invasive technique with a percutaneous pedicle screw system (Figure 3) [5]. Her radiating leg pain, motor weakness in her ankles, and symptoms of stress urinary incontinence improved markedly a few days after surgery, as did her anal sensation. Her postoperative course was uneventful except for delayed wound healing of a skin incision for placement of the left iliac screws. One week after surgery, she could use a wheel chair, and after 4 weeks, she was permitted full weight bearing, was able to walk with a cane, and was discharged. Subcutaneous injection of teriparatide 20 *μ*g daily was used postoperatively. At a 6-month follow-up examination, the patient reported no low back pain and was walking independently without pelvic complaints. CT revealed that bone union was achieved.

## 3. Discussion

The present case suggests two important clinical issues. First, although it is not common, even a minimally displaced SIF can be a cause for bladder and rectal disturbance. Second, SIF with spinopelvic dissociation and delayed neurological compromise can be treated by lumbopelvic fixation.

Sacral fracture involving neural foramina or transverse fracture is reported to be related to neurological symptoms. In SIF, an H-shaped fracture is not rare, being found with 61% of isolated SIF [6]. Rommens et al. classified this type of fracture as spinal dissociation containing a bilateral vertical fracture through the lateral mass of the sacrum with a horizontal component connecting them [2]. This type of fracture accounted for 15.1% in their case series of fragility fractures of the pelvic ring. The ventral rami of S2, S3, and S4 are distributed to the pudendal nerve, which provides sensation to the skin around the anus and perineum, and motor control of the urethral sphincter and external anal sphincter [7]. S2 to S4 nerve roots are also involved in the parasympathetic control of coordinated function of the bladder and rectum and sympathetic control of urethral and anal sphincter contraction [8]. In the present case, instability in fractured neural foramina bilaterally was considered to lead to damage of the sacral roots that resulted in neurological deficits. Diagnosis of SIF is often delayed or missed, especially in cases without obvious trauma history. Neurological deficits associated with SIF are also frequently overlooked at neurological examination because of the absence of obvious sensorimotor dermatomal distribution and delayed presentations of neurological deficits. Furthermore, stress incontinence is common in elderly women who are also at risk of SIF. Although few cases of SIF with bladder rectum dysfunction are reported, there might frequently be cases that are missed [9]. Clinicians should be aware of neurological deficits including bladder or rectum disturbance in SIF.

Surgery should be considered when patients with SIF present neurological compromise such as dysuria or leg paresthesia. However, there is no established evidence-based treatment for SIF. Conservative treatment such as bed rest and medication for pain is common. Several studies have reported surgical treatment for SIFs such as sacroplasty and percutaneous screw fixation [10–13]. Neurological compromise in SIF is often present with delayed timing, and in most cases, SIF has already been treated conservatively. Thus, to continue conservative treatment is not advocated. There is a report of 2 cases of SIF with dysuria treated with laminectomy of the sacrum. In both cases, pain in the lower limbs improved whereas dysuria and dyschezia recovered only slightly [9]. In the present case, we hypothesized that instability in the fractured neural foramina was the cause of the neurological deficits. H-shaped fractures of the sacrum with minimal displacement may only require fixation of the bilateral sacral fracture, such as sacroiliac screw osteosynthesis, sacroplasty, bridging plate osteosynthesis, or insertion of a transsacral positioning bar [2]. However, these techniques are not always suitable for SIF. With sacroiliac screw osteosynthesis, there is a risk of screw loosening due to low pullout strength [14]. Sacroplasty has the disadvantages of shared forces loaded on the cement-augmented area in the vertical fracture and delayed bony healing because of cement interposition in the fracture site [2]. Bridging plate osteosynthesis is less suitable for SIF, because the plate construct does not provide absolute stability. As a more promising approach, placement of a transsacral positioning bar is a minimally invasive approach that reduces the risk of implant loosening [15]. However, the implants used for this procedure are not available in Japan. There are few high-quality reports on the treatment of SIF, and no conclusive treatment option has been determined. Although lumbopelvic fixation may be overtreatment for the patient, we decided to repair the spinopelvic dissociation and fractured neural foramina bilaterally to acquire a rigid construct to improve the neurological symptoms, provide immediate pain relief, and enable rapid mobilization. Moreover, without neural decompression, posterior elements of the sacrum are preserved to maintain stability of the fractured sacrum. Moreover, without neural decompression, posterior elements of the sacrum are preserved to maintain stability of the fractured sacrum.

Recently, several studies have investigated the usefulness of teriparatide for SIFs [16, 17]. In addition, systematic reviews have also indicated that adjuvant drug therapy with teriparatide promotes fracture healing in patients with osteoporosis [18]. In the present case, teriparatide was used to promote fracture healing and prevent subsequent fracture.

## 4. Conclusion

Even minimally displaced SIF in patients with osteoporosis can be a cause of bowel and bladder disturbance. Lumbopelvic fixation is a treatment option for SIF with spinopelvic dissociation presenting with neurological deficits.
